# Lentivirus-Mediated Knockdown of Astrocyte Elevated Gene-1 Inhibits Growth and Induces Apoptosis through MAPK Pathways in Human Retinoblastoma Cells

**DOI:** 10.1371/journal.pone.0148763

**Published:** 2016-02-19

**Authors:** Ying Chang, Bin Li, Xiaolin Xu, Ling Shen, Haixia Bai, Fei Gao, Zhibao Zhang, Jost B. Jonas

**Affiliations:** 1 Beijing Institute of Ophthalmology, Beijing TongRen Eye Center, Beijing TongRen Hospital, Capital Medical University, Beijing Ophthalmology and Visual Sciences Key Laboratory, Beijing, China; 2 Department of Ophthalmology, Medical Faculty Mannheim of the Ruprecht-Karls-University Heidelberg, Mannheim, Germany; Kyung Hee University, REPUBLIC OF KOREA

## Abstract

**Purpose:**

To explore expression and function of astrocyte elevated gene-1 (*AEG-1*) in human retinoblastoma (RB).

**Methods:**

The expression of *AEG-1* in histological sections of human RBs and in RB cell lines was examined using immunohistochemical staining and RT-PCR and Western blotting respectively. We knocked down *AEG-1* gene levels by *AEG-1*-siRNA lentivirus transfection of human RB cell lines SO-RB50 and Y79, and using an MTT assay, we assessed the role of *AEG-1* on RB cell proliferation. The biological significance of lentivirus transfection induced *AEG-1* down-regulation was examined by assessing the apoptosis rate in the transfected RB cells by Annexin V-APC staining and flow cytometry. We additionally measured the expression of Bcl-2, Bax, cleaved-caspase-3 and caspase-3, and the phosphorylation and non-phosphorylation alternation of MAPKs.

**Results:**

*AEG-1* expression was detected to be strongly positive in the histological slides of 35 out of 54 (65%) patients with RB. *AEG-1* expression increased significantly (*P*<0.05) with tumor stage. In the RB cell lines SO-RB50, Y79 and WERI-RB1 as compared with retinal pigment epithelium cells, expression of *AEG-1* mRNA and AEG-1 protein was significantly higher. In *AEG-1*-siRNA lentivirus transfected cell cultures as compared with negative control lentivirus transfected cell cultures, levels of *AEG-1* mRNA and of AEG-1 protein (*P*<0.05) and cell growth rates (*P*<0.01) were significantly lower, and apoptosis rate (*P*<0.001), Bax/Bcl-2 ratio and cleaved-caspase-3 protein level were significantly increased. The P-ERK/ERK ratio was significantly decreased in the *AEG-1*-siRNA lentivirus transfected cell lines.

**Conclusions:**

Expression of *AEG-1* was associated with RB, in histological slides of patients and in cell culture experiments. Lentivirus transfection induced knockdown of *AEG-1* had a tumor suppressive effect, potentially by tumor cell apoptosis induction through inhibition of ERK.

## Introduction

Retinoblastoma (RB), the most common primary intraocular malignant tumor in children, has been widely studied because of the genetic mechanism leading to is development [1–5]. Previous studies proposed Knudson’s 2-hit hypothesis and revealed an inactivation of both *RB1* alleles. During the last decades, investigations enhanced our understanding of the molecular pathogenesis of RB and proposed a multi-step process for the progression of normal retinal cells to RB cells [1,6,7]. The details of this multi-step development have remained elusive so far [8].

In 2002, astrocyte elevated gene-1 (*AEG-1*) was cloned as a neuropathology-associated gene in primary human fetal astrocytes and has in the meantime emerged as an important oncogene that is markedly overexpressed in multiple types of human cancer [9–12]. Numerous studies established a functional role of *AEG-1* in the pathogenesis and progression of tumors, the regulation of apoptosis, and the induction of metastasis by activating diverse oncogenic signaling pathways, such as NF-kappaB, Ha-ras, PI3K/AKT, MAPK, and WNT pathways [12–14]. The mitogen activated protein kinases (MAPKs) cascades, activated by various cellular stress factors and growth factors, are major signaling transduction molecules in the process of apoptosis. The role the MAPKs play in the cascade of apoptosis varies in dependence of the kind of stimulus and type of cell [15]. In human RB cells, Yunoki and colleagues found that silencing of BCL2-associated athanogene 3 enhanced the effects of HT-induced apoptosis by increasing phosphorylation of ERK (Extracellular-signal Regulated Kinase) [16]. Min and associates showed that 2-Methoxyestradiol induced apoptosis via activation of p38 MAPK and ERK [17]. The expression and functional role of *AEG-1* in human RB has not been examined yet and the role of the MAPK pathways in the process of apoptosis in RB cells has remained controversial. We therefore conducted this study to explore the expression of *AEG-1* mRNA and AEG-1 protein in three RB cell lines and in human RB samples, and then to construct a lentivirus-mediated knockdown of *AEG-1* to downregulate its expression in the human retinoblastoma Y79 and SO-RB50 cell lines in vitro. In controlled transfected RB cell colonies, we then examined the effect of *AEG-1* silencing on the proliferation and apoptosis of RB cells and explored the potential mechanism.

## Methods

### Tissue samples and immunostaining

Histological sections of human eyes enucleated due to retinoblastoma underwent immunostaining of *AEG-1* using the anti-AEG-1 antibody (Abcam Co, Milton, UK). The study was approved by the ethics committee of Tongren Hospital and followed the Declaration of Helsinki. Due to the retrospective recruitment of the tissue samples, the ethics committee waived the necessity of obtaining a written informed consent from the children or their parents. For the study purposes, the patient tissue samples were de-identified and analyzed anonymously.

Negative controls were performed using non-specific immunoglobulin. Two pathologists not involved in the present study evaluated the immunostaining under masked conditions. The degree of expression was graded according to the percentage of positive cells and staining intensity: negative expression (0–20% positive cells), weakly positive expression (20–50% positive cells) and strongly positive expression (50–100% positive cells). The scale was determined according to the average number of positive cells in five arbitrary fields of all slides. For statistical analysis, the “negative expression” group and the “weakly positive expression” group were combined to form the “negative AEG-1 expression” group. We then compared clinicopathological features of the RBs between the group with high-level AEG-1 expression ("strongly positive") and the group with “negative AEG-1 expression.

### Cell culture

The human retinoblastoma cell lines Y79, SO-RB50 and WERI-RB1 and retinal pigment epithelium (RPE) cells were obtained from the department of pathology of the Zhongshan Ophthalmic Center, Sun Yat-sen University and the Chinese University of Hong Kong. The RB cells were maintained in RPMI-1640 medium (Hyclone Laboratories Inc., Logan, Utah, U.S.A) supplemented with 10% fetal bovine serum and 1% penicillin-streptomycin in a humidified atmosphere of 5% CO2/95% air at 37°C. The culture medium was replaced every 3 days.

### Lentivirus vectors for *AEG-1* small interfering RNA

Lentivirus vectors for *AEG-1* small interfering RNA were used to examine the function of *AEG-1*. A third generation of the self-inactivating lentiviral vector containing a CMV (cytomegalovirus) promoter-driven eGFP (enhanced green fluorescence protein) reporter was purchased from Shanghai Genechem Co., Ltd. (Shanghai, China). pGCL-GFP-Lentivirus was used to express small interfering RNAs (siRNAs) targeting the *AEG-1* ORF sequence (Genbank No. NM_178812) (*AEG-1*-siRNA lentivirus). A non-targeting sequence was used as a negative control (NC) lentivirus. The *AEG-1*-siRNA sequence was 5’- AACAGAAGAAGAAGAACCGGA -3’, and the NC sequence was 5’-TTCTCCGAACGTGTCACGT -3’. The sequences were cloned into the pGCSIL-GFP (GeneChem Co., Shanghai, China) to generate the lentiviral vectors.

### Y79 and SO-RB50 cells were infected with *AEG-1*-siRNA lentivirus

The human RB cell lines Y79 and SO-RB50 were infected with *AEG-1*-siRNA lentivirus vectors (KD (knockdown) group) and NC lentivirus vectors (NC group). Non-transfected cells were included as control (Con group). After 3 days of transfection, GFP expression was assessed by fluorescence microscopy. After 5 days of transfection, cells were collected to determine the knock-down effect by quantitative real-time PCR (RT-PCR) and Western blot analysis.

### Quantitative real-time polymerase chain reaction (RT-PCR) analysis

Total RNA was extracted from the cells using the Trizol reagent (Invitrogen Co., Carlsbad, CA, USA). The reverse transcription and the PCR amplification reactions were performed according to the M-MLV reverse transcriptase protocol (Promega Biotech Co. Ltd, Beijing, China). RT-PCR was carried out using an ABI PRISM 7900 Sequence Detection System (Applied Biosystems Co., Carlsbad, CA, USA). The primers for RT-PCR were: human *AEG-1* (121bp): 5’-TGACTTCAACAGCGACACCCA-3’ forward, 5’-CACCCTGTTGCTGTAGCCAAA-3’ reverse; human *GADPH* (111bp): 5’-AAGCAGTGCAAAACAGTTCACG-3’ forward, 5’-GCACCTTATCACGTTTACGCT-3’ reverse. The threshold cycle (Ct values), which was the cycle number at which the amount of amplified gene of interest reached a fixed threshold, was subsequently determined. The *AEG-1* mRNA levels was normalized to human *GADPH* levels and calculated with the 2^-∆∆Ct^ method [18].

### Western blot analysis

The Western blot analysis and the whole-cell lysates were carried out as described in detail previously [19]. The blots were probed with *AEG-1* (Abcam Co., Milton, UK), Bcl-2, Bax, cleaved-caspase-3, caspase-3, phospho-ERK, ERK, phospho-JNK, JNK, phospho-p38 MAPK, p38 MAPK (Cell Signaling Technology Co., Danvers, MA, USA) and GAPDH antibodies (ImmunoWay Co., Newark, DE, USA). The protein expression was visualized after extensive washing using the ECL (enhanced chemiluminescence) advanced detection kit (GE Healthcare Co., Buckinghamshire, UK) and quantified with the Quantity One software (Bio-Rad Laboratories Inc., Hercules, CA, USA).

### Cell viability assay

Cell viability was determined using the MTT method, which is based on the reduction of tetrazolium salt by mitochondrial dehydrogenase in viable cells. Following transfection as described above, the cells were transferred to 96-well plates and seeded at a volume of 90 μl of cell suspension (3000 cells/well). The absorbance at 490 nm was measured read using a spectrophotometric plate reader. The procedure was repeated every 24 hours during a 5-day period. Cell survival rates were measured at 3 time points of the cell growth curve through the log phase of growth for each cell line.

### Cell apoptosis analysis

At the seventh day after transfection, cell apoptosis was examined by flow cytometry after the cells were stained with 100 μl cell suspension containing 5 μl Annexin V-APC at room temperature in the dark for 10–15 min. All experiments were performed in triplicate.

### Statistical analysis

Statistical analysis was performed using a commercially available statistical software program (SPSS 22.0 for Windows, IBM-SPSS Inc., Chicago, IL, USA). Mean ± standard deviations and standard error of the measurements obtained in the three independent experiments were presented. Analysis of the statistical significance of differences between groups was carried out by a one-way analysis of variance (ANOVA). To assess an association between the *AEG-1* expression and clinicopathological features of the RBs, we performed a regression analysis. A *P*-value <0.05 was considered to indicate statistical significance.

## Results

### Increased expression of AEG-1 correlates with clinicopathological features of RB

**Fig 1:**
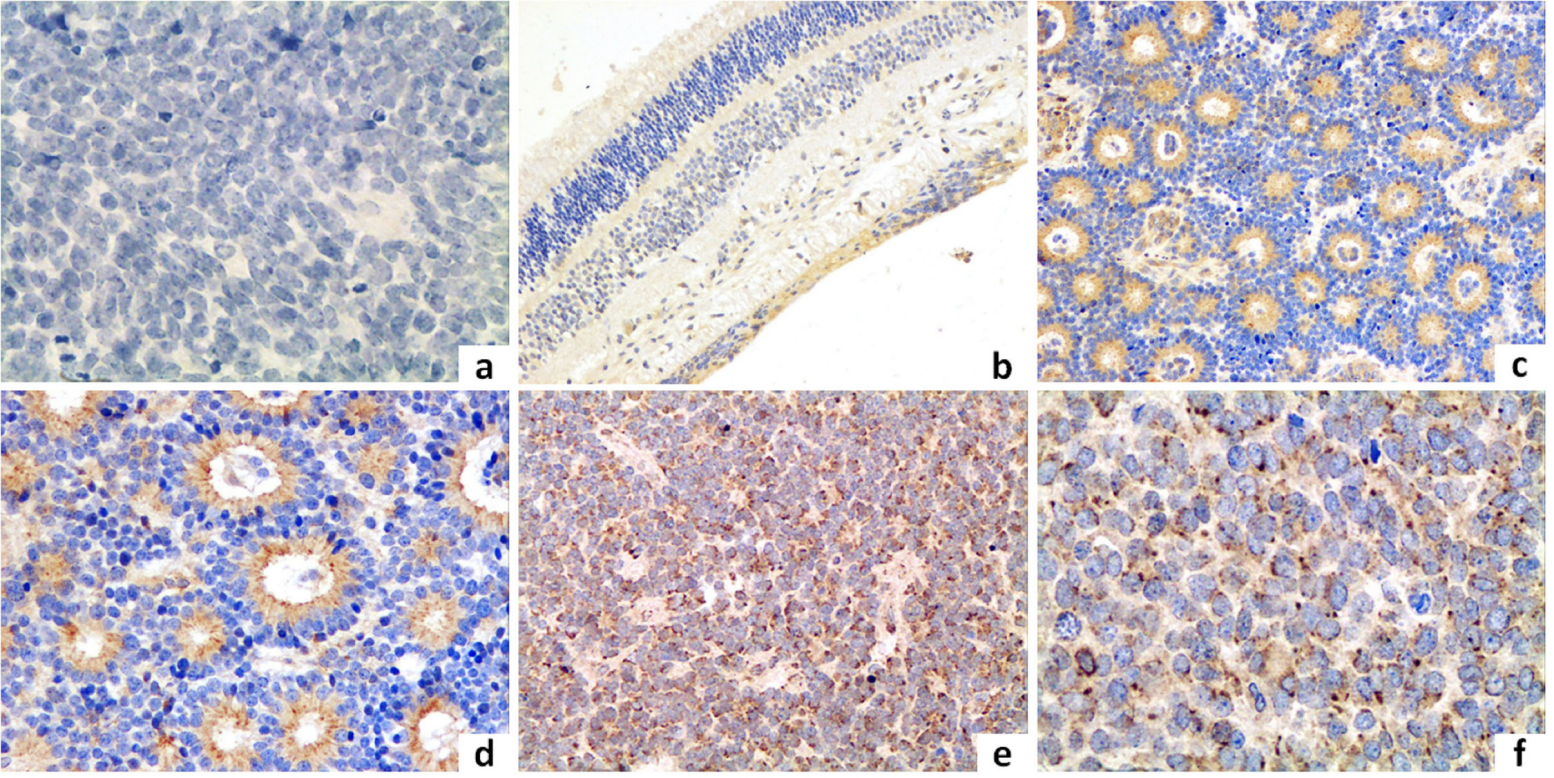
Immunohistochemistry in retinoblastoma tissues. 1a: Negative control stained with non-specific immunoglobulin; 1b: Immunostaining of AEG-1 in the relatively normal retinal tissue of the peri-tumor region (original magnification, ×100). 1c, d: Immunostaining of AEG-1 in well-differentiated specimens (original magnification, c: ×200; d: ×400); 1e, f: Immunostaining of AEG-1 in poorly-differentiated specimens (original magnification e: ×200; f: ×400).

To investigate whether the AEG-1 protein was overexpressed in clinical samples of RB, we immunohistochemically examined paraffin-embedded histological sections of 54 eyes which had been enucleated because of the RBs (Fig 1). *AEG-1* expression was present predominantly in the perinuclear region and was detected in 49 of 54 (91%) patients, with a low-level *AEG-1* expression in 19 (35%) patients and a high-level *AEG-1* expression in 35 cases (65%). The high-level *AEG-1* expression was significantly (*P* = 0.035) higher in tumors of stage “E” than in tumor of stage “D”, and it was higher in patients with optic nerve invasion as in patients without optic nerve involvement (*P* = 0.02). The AEG-1 protein expression was not significantly associated with gender (*P* = 0.51), right or left eyes (*P* = 0.31), ages (*P* = 0.58), amount of choroid tumor invasion (*P* = 0.24), and occurrence of a tumor invasion into the anterior segment (*P* = 0.58) (Table 1).

**Table 1:** Correlation between high-level *AEG-1* expression and the clinicopathological features of retinoblastoma [n(%)].

Clinicopathological Features	Patients	*AEG-1* Positive Expression	χ^2^	*P*-Value
Gender			0.43	0.51
Men	28 (52)	17 (61)		
Female	26 (48)	18 (69)		
Laterality			1.05	0.31
Right	29 (54)	17 (59)		
Left	25 (46)	18 (72)		
Age (Years)			0.31	0.58
< 3	48 (89)	30 (63)		
≥3	6 (11)	5 (83)		
Clinical Stage			4.46	0.035
Stage D	21 (39)	10 (48)		
Stage E	33 (61)	25 (76)		
Differentiation			0.38	0.54
Poor	41 (76)	28 (68)		
High	13 (24)	7 (54)		
Invasion of Optic Nerve			5.68	0.02
None / Prelaminar / Laminar	34 (63)	18 (53)		
Postlaminar / Resected Margin	20 (37)	17 (85)		
Choroidal Invasion			1.39	0.24
None / Focal	42 (78)	25(59.52)		
Massive	12 (22)	10(83.33)		
Involvement of Anterior Segment			0.31	0.58
None	48 (89)	30 (63)		
Yes	6 (11)	5 (83)		

### Up-regulation of AEG-1 in the human RB cell lines

**Fig 2:**
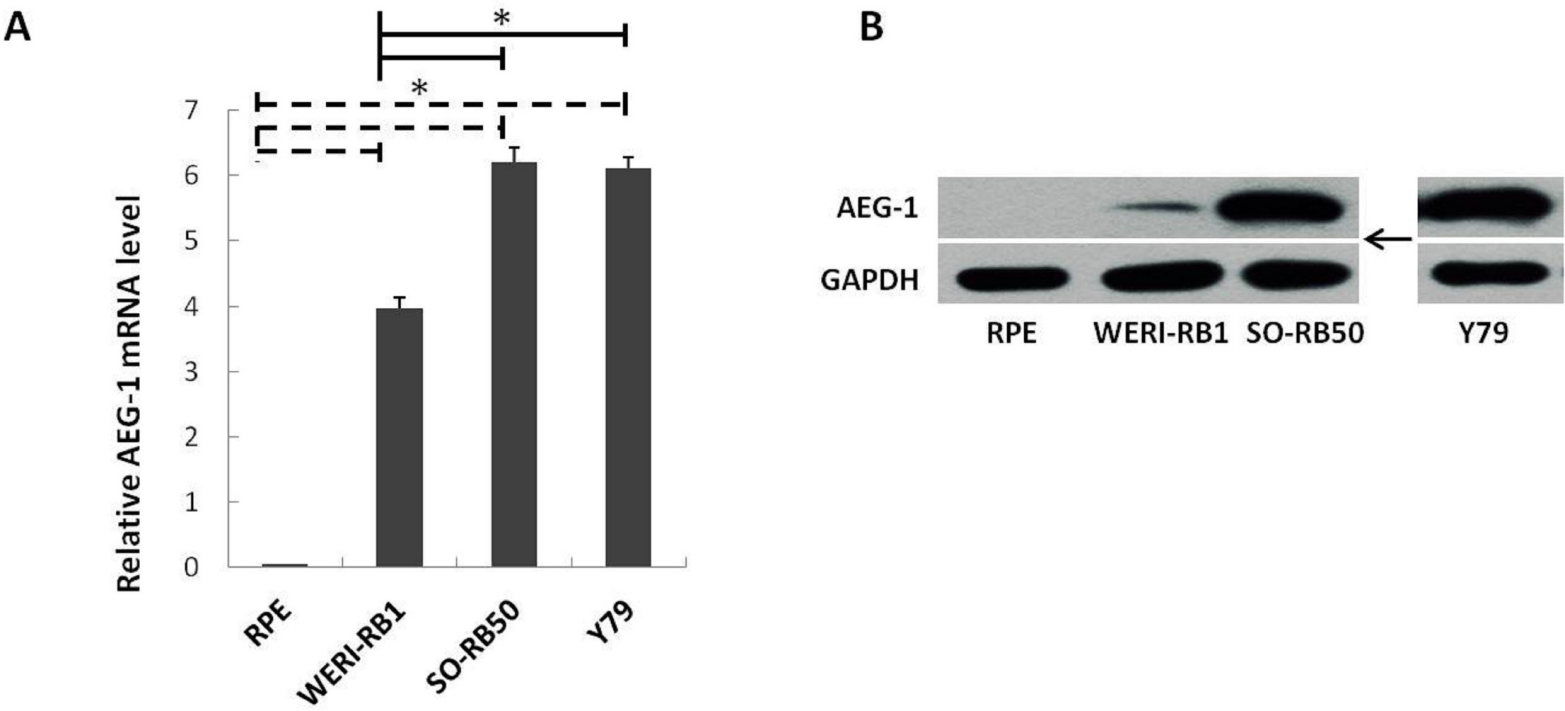
*AEG-1* expression in retinoblastoma (RB); 2A: Equivalent amounts of RNA were analyzed by RT-PCR in three RB cell lines (WERI-RB1, SO-RB50, Y79) and in retinal pigment epithelium cells (RPE) as controls. Results were presented as mean ± standard error, obtained in three independent experiments; * indicated the statistical difference between the retinal pigment epithelium cells (RPE) and each of the RB cell lines and between the RB cell lines (*P*<0.05). 2B: AEG-1 and GAPDH protein levels in untreated cell lines.

To determine the relative expression levels of *AEG-1* in the RB cells, equivalent quantities of RNA and protein were analyzed by RT-PCR and Western blotting, respectively. The mRNA and protein expression levels of *AEG-1* were significantly higher in the three RB cell lines as compared with the RPE cells (Fig 2A and 2B). Within the group of RB cell lines, the expression level was significantly higher in the SO-RB50 cell line and Y79 cell line than in the WERI-RB1 cell line (Fig 2A and 2B).

### Lentivirus-mediated knock-down of *AEG-1* gene in the SO-RB50 and Y79 cell lines

**Fig 3:**
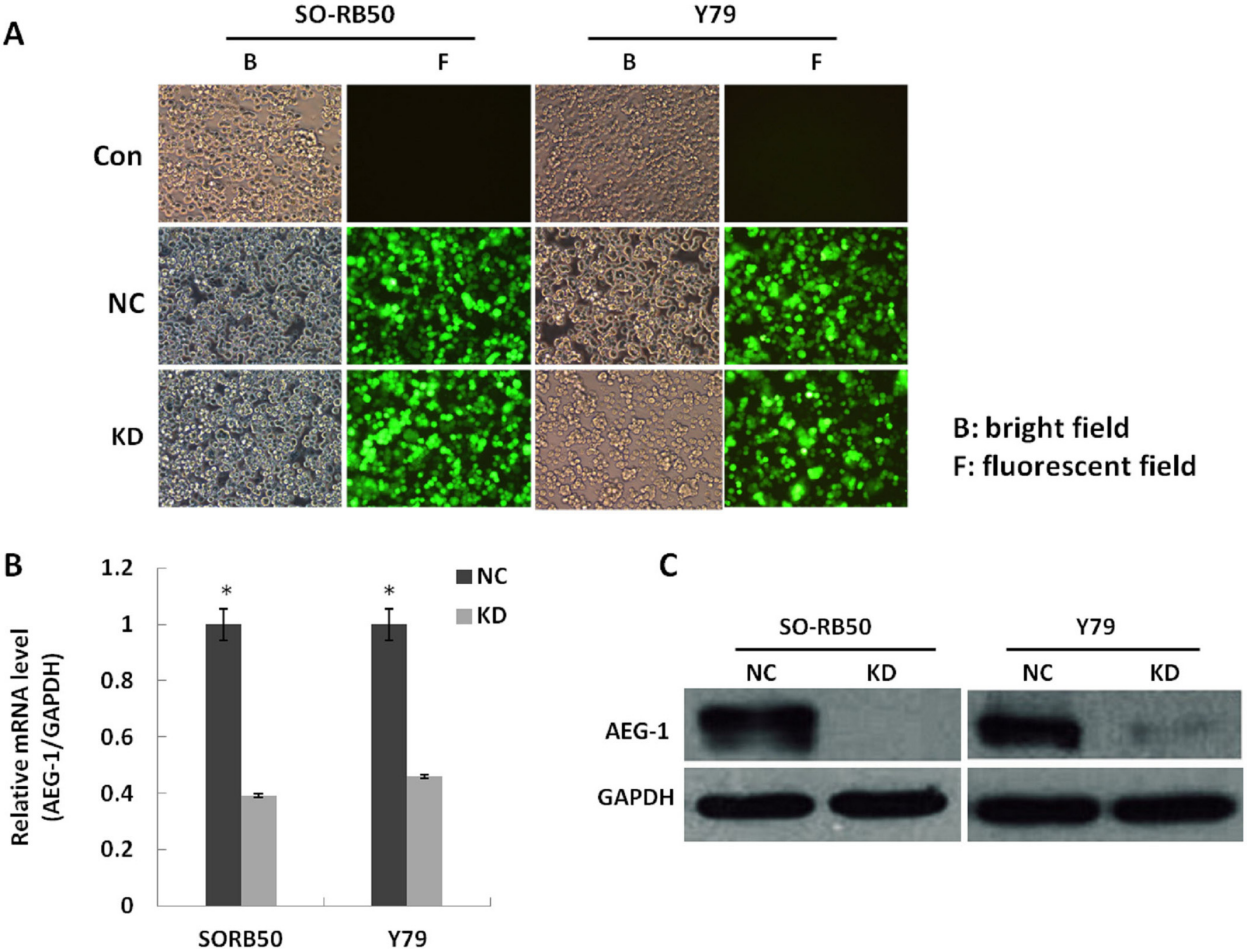
Lentivirus-mediated knock-down of *AEG-1* gene in human retinoblastoma (RB) cell lines SO-RB50 and Y79; 3A: Human RB cell lines Y79 and SO-RB50 transfected with *AEG-1*-siRNA (KD groups) or with NC lentivirus and non-transfected cells were examined by fluorescent microscopy and light microscopy at the 3rd day after infection. More than 80% of RB cells expressed GFP. 3B: *AEG-1*-siRNA lentivirus transfected cultures had significantly lower levels of *AEG-1* mRNA compared to levels in cultures transfected with NC lentivirus (*P<0.05). 3C: The protein expression level of AEG-1 decreased significantly in the KD groups compared to that in the NC groups. For comparison, the GAPDH protein did not vary significantly between the groups.

To investigate the biological significance of a down-regulation of *AEG-1* in RB, we knocked down *AEG-1* gene levels with the *AEG-1*-siRNA lentivirus in the human RB cell lines SO-RB50 and Y79. At 3 days after transfection, GFP expression was assessed by fluorescence microscopy for both A*EG-1*-siRNA lentivirus and NC lentivirus (Fig 3A). The negative control cells and the non-transfected cells did not differ significantly indicating that the transfection process had not influenced the cell growth. The efficacy of the lentivirus-mediated knock-down on the concentrations of *AEG-1* mRNA and AEG-1 protein in the SO-RB50 and Y79 cell lines was determined at the 5^th^ day after transfection by RT-PCR and Western blotting. The *AEG-1*-siRNA lentivirus infected cell cultures as compared with the cell cultures infected with the NC lentivirus had significantly (*P*<0.05) lower levels of *AEG-1* mRNA (Fig 3B). The protein expression level of *AEG-1* decreased significantly in the *AEG-1 k*nockdown groups compared to that in the negative control groups. For comparison, the GAPDH protein did not vary significantly between the groups (Fig 3C).

### Knockdown of *AEG-1* inhibits proliferation and promotes apoptosis in human RB cells

**Fig 4:**
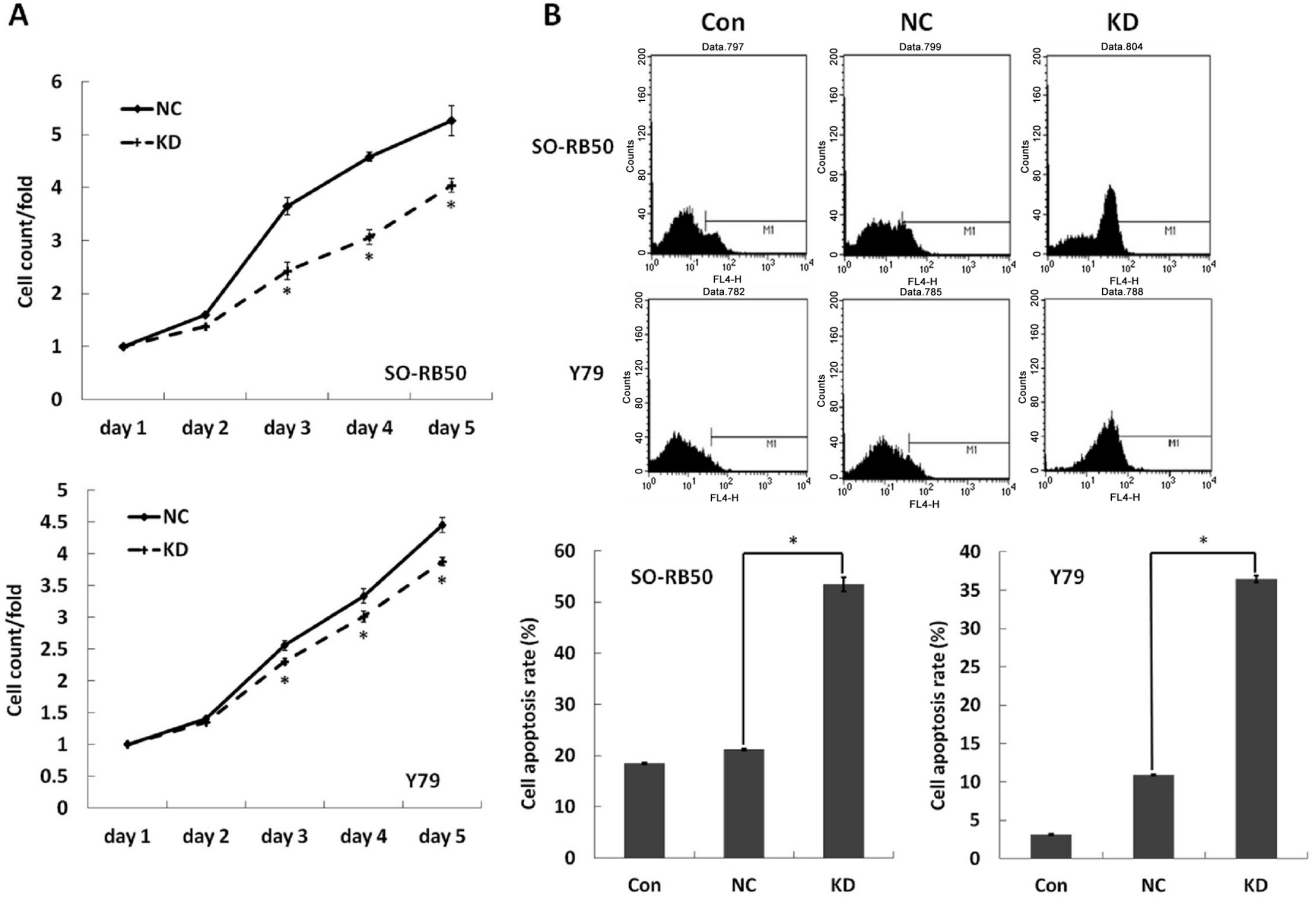
Effect of *AEG-1* knockdown on the proliferation and apoptosis in human retinoblastoma (RB) cell lines SO-RB50 and Y79. The data were expressed as mean ± standard error, obtained in three independent experiments. 4A: Cell proliferation was examined by the MTT assay every day for 5 days. Knock down of *AEG-1* showed a significant decline in cell growth rate compared to NC group (*P<0.05). 4B: Cell apoptosis was determined by Annexin V-APC staining and followed by flow cytometry. It was significantly increased in the *AEG-1* Knockdown group compared to the NC group (*P<0.05).

To detect the role of *AEG-1* on the RB cell proliferation, we examined daily the effect of the *AEG-1* knockdown on RB cell growth using the MTT assay for 5 days. Cell growth rate was defined as: cell count of n^th^ day/cell count of 1^st^ day, where n = 2,3,4,5. A significant inhibition of the cell growth rate was found in the *AEG-1* knockdown groups of the SORB50 cell line and Y79 cell line as compared to the negative control groups at the 3^th^, 4^th^ and 5^th^ day (SORB50: *P*_3th day_ = 0.001, *P*_4th day_<0.001, *P*_5th day_ = 0.002; Y79: *P*_3th day_ = 0.009, *P*_4th day_ = 0.015, *P*_5th day_ = 0.002) (Fig 4A).

To test whether the *AEG-1* expression affected the RB cell apoptosis, we evaluated the intensity of apoptosis by Annexin V-APC staining and flow cytometry. Cell apoptosis was significantly increased in the *AEG-1 k*nockdown groups as compared to the negative control groups (SORB50: NC 21.21 ± 0.17% vs. KD 53.45 ±1.37%, *P*<0.001; Y79: NC 10.91 ± 0.06% vs. KD 39.46 ± 0.42%; *P*<0.001) (Fig 4B).

### Effects of *AEG-1* knockdown on Bcl-2 family and caspase-3 levels in RB cells

**Fig 5:**
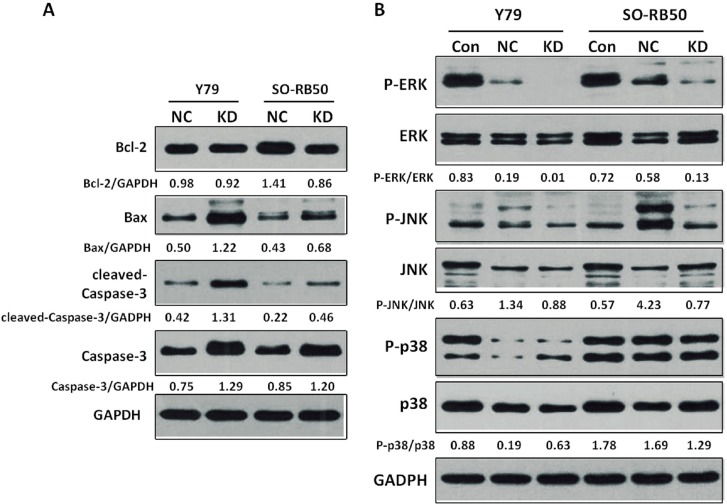
5A: Effects of *AEG-1* knockdown on expression of apoptosis-related proteins in human retinoblastoma (RB) cells. Cell lysates were electrophoresed and Bcl-2, Bax, cleaved-caspase-3 and caspase-3 were detected by Western blotting analysis with the corresponding antibodies. 5B: Regulation of MAPKs in *AEG-1* knockdown human RB cells. Equal amounts of cell lysates were electrophoresed and JNK, ERK, and p38 and their phosphorylated expression form were detected by Western blotting analysis with corresponding antibodies.

To assess the apoptotic effects of the *AEG-1* knockdown on the RB cells, we examined the expression levels of apoptosis regulatory proteins Bcl-2, Bax and caspase-3. The level of Bcl-2 protein was down-regulated in the SO-RB50 knockdown cell line in comparison to the negative control group, while it did not differ significantly between the Y79 knockdown cell line and the negative control group. The level of Bax protein was up-regulated in the Y79 knockdown cell line and SO-RB50 knockdown cell line. The Bax/Bcl-2 ratio was elevated in both cell lines (Y79, SO-RB50) infected with the *AEG-1*-siRNA lentivirus (Y79 cell line: KD 1.33 vs. NC 0.51; SO-RB50 cell line: KD 0.79 vs. NC 0.3). The level of cleaved-caspase-3 protein was markedly enhanced both in the Y79 knockdown cell line and the SO-RB50 knockdown cell line. For comparison, the GADPH protein did not differ markedly between the groups (Fig 5A).

### Knockdown of AEG-1 decrease the expression of ERK and increases the expression of JNK in human RB cells

To examine whether MAPKs were involved in the growth inhibition and induced apoptosis of the RB cells after lentivirus transfection induced silencing of the *AEG-1* gene, we assessed the phosphorylation and non-phosphorylation alternation of MAPKs. The knockdown of *AEG-1* significantly decreased the P-ERK/ERK ratio in both the Y79 cell line and the SO-RB50 cell line (Fig 5B). The P-p38/p38 ratio was up-regulated in the knockdown Y79 cell line and it was down-regulated in the SO-RB50 cell line.

## Discussion

Our study revealed an expression of *AEG-1* in the histological slides of 91% of the patients with RB, with a higher expression in the eyes with a higher stage of the tumor (Fig 1). As a corollary, AEG-1 protein was significantly more expressed in the RB cell lines than in the RPE cell line suggesting that expression of *AEG-1* was associated with RB (Fig 2A and 2B). After silencing of *AEG-1* by lentivirus transfection, the growth rate of RB cell lines was significantly reduced and the rate of cell apoptosis was significantly increased (Fig 4A and 4B). Correspondingly, the expression levels of apoptosis inducing protein Bax was up-regulated, the Bax/Bcl-2 ratio was elevated, and the level of caspase-3 protein was markedly increased in the RB cell lines (Fig 5A). With respect to MAPKs, the knockdown of *AEG-1* significantly decreased the P-ERK/ERK ratio in both RB cell lines (Fig 5B). These results indicated that an increased expression of *AEG-1* was associated with RB, and that its knockdown had a tumor suppressive effect, potentially by an induction of induced tumor cell apoptosis through inhibition of ERK.

The expression level of *AEG-1* was significantly higher in the SO-RB50 cell line and in the Y79 cell line than in the WERI-RB1 cell line (Fig 2). It was in accordance with the differences in the characteristics of the original tumors which served as sources for the cell lines. The SO-RB50 cell line was derived from the tumor of a 53-day-old Chinese girl with optic nerve invasion. The Y79 retinoblastoma cell line was derived from the tumor of a 2.5-year-old Caucasian girl with a strong maternal history of RB. The original tumor was mostly undifferentiated and showed intraocularly an invasive growth [20]. The WERI-RB1 cell line came from the tumor of a 1-year-old Caucasian girl with no family history of RB. This tumor was only partially undifferentiated, and only some portions of the optic nerve head anterior to the lamina cribrosa were invaded by tumor cells [21]. Examining animal models, Chévez-Barrios and colleagues showed that the Y79 RB model in animals was similar to the situation seen in patients with RBs demonstrating an invasive and metastatic disease, whereas the WERI-RB1 model more closely resembled non-metastatic human RBs [22].

The results of our study are in agreement with previous investigations which were focused on other types of tumors [23–27]. Aberrant *AEG-1* expression has been observed in multiple types of tumors including gliomablastoma, neuroblastoma, oligodendroglioma and meningioma. *AEG-1* has been shown to be involved in the coordination of diverse signal-transduction pathways allowing the tumor cells to proliferate, evade from apoptosis, invade and to form metastases [28]. The high frequency of *AEG-1* overexpression in diverse cancers with poor prognosis may suggest that *AEG-1* may be used as a potential diagnostic or prognostic biomarker for cancer [12]. Correspondingly, Yoo and colleagues suggested that *AEG-1* now emerging as an important oncogene in a wide array of cancers may become a molecular target for a future”pan-cancer” therapy [14]. Our study extended the findings obtained previously on other tumors into the field of RB.

A malignant tumor is characterized by an uncontrolled proliferation and dysregulated apoptosis [29]. The intrinsic mitochondrial-related apoptotic pathway, leading to the permeabilization of the outer mitochondrial membrane and the release of proteins from the mitochondrial intermembrane space is regulated by the Bcl-2 family [30]. Following an apoptotic stimulus, activated pro-apoptotic proteins (such as Bax) reach the outer mitochondrial membrane, open the membrane pores and allow the release of cytochrome c into the cytosol. In contrast, anti-apoptotic proteins (such as Bcl-2) on the outer mitochondrial membrane block the membrane pores and maintain the integrity of the mitochondrial membrane [30]. The expression of Bcl-2 and Bax proteins have been examined in RB [31–33]. In the present study, the down-regulation of Bcl-2 protein and up-regulation of Bax protein may have been related with an increased apoptosis induced by silencing the *AEG-1* gene. Correspondingly, the Bax/Bcl-2 ratio as a determining factor in regulating the apoptotic process increased in the RB cell lines of our study after knockdown of *AEG-1* [34]. As a corollary, cleaved-caspase-3 which is considered to function downstream of the cytochrome c release was markedly activated in *AEG-1* knockdown RB cells, also suggesting that silencing of *AEG-1* may induce apoptosis through the mitochondrial pathway [30].

Mitogen activated protein kinases (MAPKs) are a family of serine/threonine protein kinases widely conserved among eukaryotes. They include three sequentially activated kinase complexes, the extracellular regulated kinase (ERK), the c-Jun NH_2_-terminal kinase (JNK) and the p38 MAPK, which are substrates for phosphorylation by MAPK kinases [15,35]. The signaling cascades play a crucial role in the regulation of cell proliferation and survival. In particular, the pharmacological modulation of MAPK signals affects the apoptotic response to anti-tumor agents [36]. MAPKs may have biphasic roles dependent upon cell type, stimuli, intensity and duration of activation, and they crosstalk with other signaling pathways [37]. The ERK pathways are involved in the regulation of meiosis and mitosis, which can be activated by the proto-oncogene *Ras* in many human tumors. It contributes to the increased proliferation rate of tumor cells [15]. The ERK pathways have also been implicated in generating anti-apoptotic signals in HaCaT cells by inhibiting caspase-3 activation following ultraviolet A-irradiation [38]. JNKs are important for the control of both apoptosis and survival signaling [39]. It has been suggested that JNKs can phosphorylate and inactivate the anti-apoptotic proteins Bcl-2 and Bcl-x_L_. Alternatively, JNKs can induce apoptosis by directly phosphorylating and activating pro-apoptotic BAD and Bim [37]. The p38 MAPKs are key regulators of inflammatory cytokine expression, and also regulate apoptosis by regulating the activities of the Bcl-2 family protein [15,40]. Recent findings indicated a requirement for a correct balance between ERK, JNKs and p38 signaling pathways to ensure appropriate regulation of apoptosis in response to a variety of cellular injuries [41]. Aberrant activation of MAPKs by *AEG-1* was detected in cancers. Yoo and colleagues found that that *AEG-1* activated MAPK pathways, especially ERK and p38 MAPK, in human hepatocellular carcinoma cells that promoted invasion and anchorage-independent growth [13]. Another recent study showed that knockdown of *AEG-1* could enhance the sensitivity of breast cancer cells to a novel ATP noncompetitive inhibitor of MAP/ERK kinas3 [42]. In our study, the role of MAPKs in *AEG-1* knockdown induced apoptosis in RB cells was examined. Interestingly, the silencing of *AEG-1* in the RB cells suppressed the activities of ERK to promote RB cells apoptosis. These results indicated that *AEG-1* might be important for the constitutive activation of MAPK in RB cells leading to enhanced cancer cell viability. The paradox ratio of P-p38/p38 between an increase in the Y79 RB cell line and a decrease in the SO-RB50 RB cell line may be explained by differences between cell lines in their origin, growth characteristics and morphologic structure.

Limiting factors of our investigation should be mentioned. First, the cell culture part of our investigation was conducted in unnatural conditions so that results obtained could not be directly transferred to a clinical situation. This limitation holds true for any cell culture investigation and is not valid for the part of our study examining the histologic slides of human eyes. Second, it remained unclear why in Fig 5B the protein expression was different between the control lane and the negative siRNA lane. Third, WERI-RB1 cells were not included in the experiments beyond those described in Fig 2. The reason was that the WERI-RB1 cell line had a slow growth rate and poor growth state and did not tolerate the virus transfection in the setting of our study, although this cell line had been used by other researchers for transfection studies [43]. This difference between the WERI-RB1 cell line on one hand and the two other cell lines in our study may also have been the reason for the lower *AEG1* expression in WERI-Rb1 cells as compared to the other two cell lines (Fig 2). Fourth, we tried to investigate the effects of *AEG-1* on the migration and invasion ability of the RB cells by transwell matrix penetration assay, however, the suspension cells of the Y79 cell line and the SO-RB50 cell line were not found to show an invasion ability. This was an important question since these cell lines had also been used for invasion assay in other studies [44].

In conclusion, our study demonstrated an overexpression of *AEG-1* in RB cell lines and tissue from RB tumors from patients. This clinical expression of *AEG-1* was positively related to the clinical stage of the tumors. Silencing the *AEG-1* gene by lentiviral vector transfection resulted in a growth inhibition and promotion of apoptosis in cultured RB cells. Correspondingly, knockdown of *AEG-1* increased the Bax/Bcl-2 ratio and activated caspase-3 in RB cells, potentially mediated through the ERK signaling pathway. The results indicate that *AEG-1* may play a role in the development and progression of RB. *AEG-1* may potentially be a novel genetic biomarker to serve as a molecular target for new anticancer therapies to prevent RB cell progression.
