# Correction: Lentivirus-Mediated Knockdown of Astrocyte Elevated Gene-1 Inhibits Growth and Induces Apoptosis through MAPK Pathways in Human Retinoblastoma Cells

**DOI:** 10.1371/journal.pone.0223818

**Published:** 2019-10-14

**Authors:** Ying Chang, Bin Li, Xiaolin Xu, Ling Shen, Haixia Bai, Fei Gao, Zhibao Zhang, Jost B. Jonas

There is an error in the text of the Methods section in [1] reporting the primers used for RT-PCR. The correct text is: The primers for RT-PCR were: human *AEG-1* (111bp): 5’-AAGCAGTGCAAAACAGTTCACG-3’ forward, 5’-GCACCTTATCACGTTTACGCT-3’ reverse; human GADPH (121bp): 5’-TGACTTCAACAGCGACACCCA-3’ forward, 5’-CACCCTGTTGCTGTAGCCAAA-3’ reverse.

The authors provide the following additional methodological information: We collected 54 clinical samples of retinoblastoma from the Ophthalmic Department of Beijing Tongren Hospital from March to December 2012. All tissues were fixed by 10% formaldehyde (volume fraction), embedded by conventional paraffin, stained by Haematoxylin-Eosin. The selected participants were not treated with chemotherapy or radiotherapy before surgery, and spontaneous retrograde atrophy eyeball was excluded. According to International Retinoblastoma Classification, the participants were divided into clinical phase D and phase E [2].

Fig 5A and 5B each incorrectly show a single shared GAPDH panel for the western blot experiments. The western blot for each protein was carried out with its own GAPDH loading control. A revised Fig 5 is provided in which the correct corresponding GAPDH loading control panels from the original experiment are shown. Please see the correct Fig 5 here.

**Fig 5:**
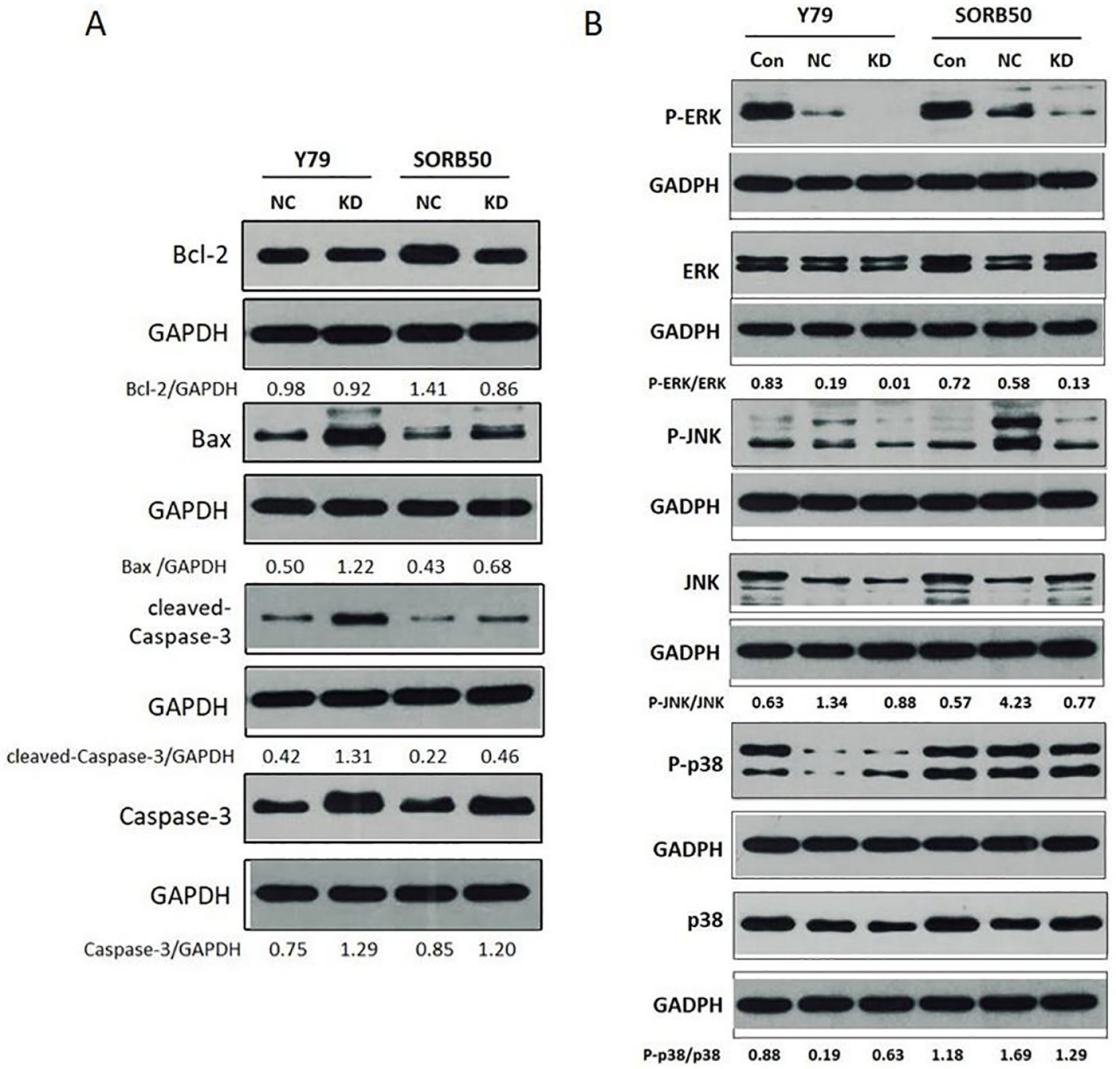
5A: Effects of AEG-1 knockdown on expression of apoptosis-related proteins in human retinoblastoma (RB) cells. Cell lysates were electrophoresed and Bcl-2, Bax, cleaved-caspase-3 and caspase-3 were detected by Western blotting analysis with the corresponding antibodies. 5B: Regulation of MAPKs in AEG-1 knockdown human RB cells. Equal amounts of cell lysates were electrophoresed and JNK, ERK, and p38 and their phosphorylated expression form were detected by Western blotting analysis with corresponding antibodies.

The available underlying data for the article are provided as Supporting Information in this Correction. Please note the individual-level clinical data for the retinoblastoma patient samples is no longer available.

## Supporting information

S1 File Available underlying data. (ZIP)

## References

[1] Chang Y, Li B, Xu X, Shen L, Bai H, Gao F, et al (2016) Lentivirus-Mediated Knockdown of Astrocyte Elevated Gene-1 Inhibits Growth and Induces Apoptosis through MAPK Pathways in Human Retinoblastoma Cells. PLoS ONE 11(2): e0148763 10.1371/journal.pone.0148763 26894431 PMC4760765

[2] Shields CL, Mashayekhi A, Au AK, Czyz C, Leahey A, Meadows AT, et al The International Classification of Retinoblastoma predicts chemoreduction success. Ophthalmology. 2006; 113(12): 2276–2280. 10.1016/j.ophtha.2006.06.018 16996605

