# Supplementary material for: Correction: Lentivirus-Mediated Knockdown of Astrocyte Elevated Gene-1 Inhibits Growth and Induces Apoptosis through MAPK Pathways in Human Retinoblastoma Cells
Source: PLoS One. 2019 Oct 14;14(10):e0223818. doi: 10.1371/journal.pone.0223818 (PMC6791552; doi:10.1371/journal.pone.0223818)
Supplement: S1 File — (ZIP) [file pone.0223818.s001.zip › Fig 5A_Image.pptx]

## Slide 1
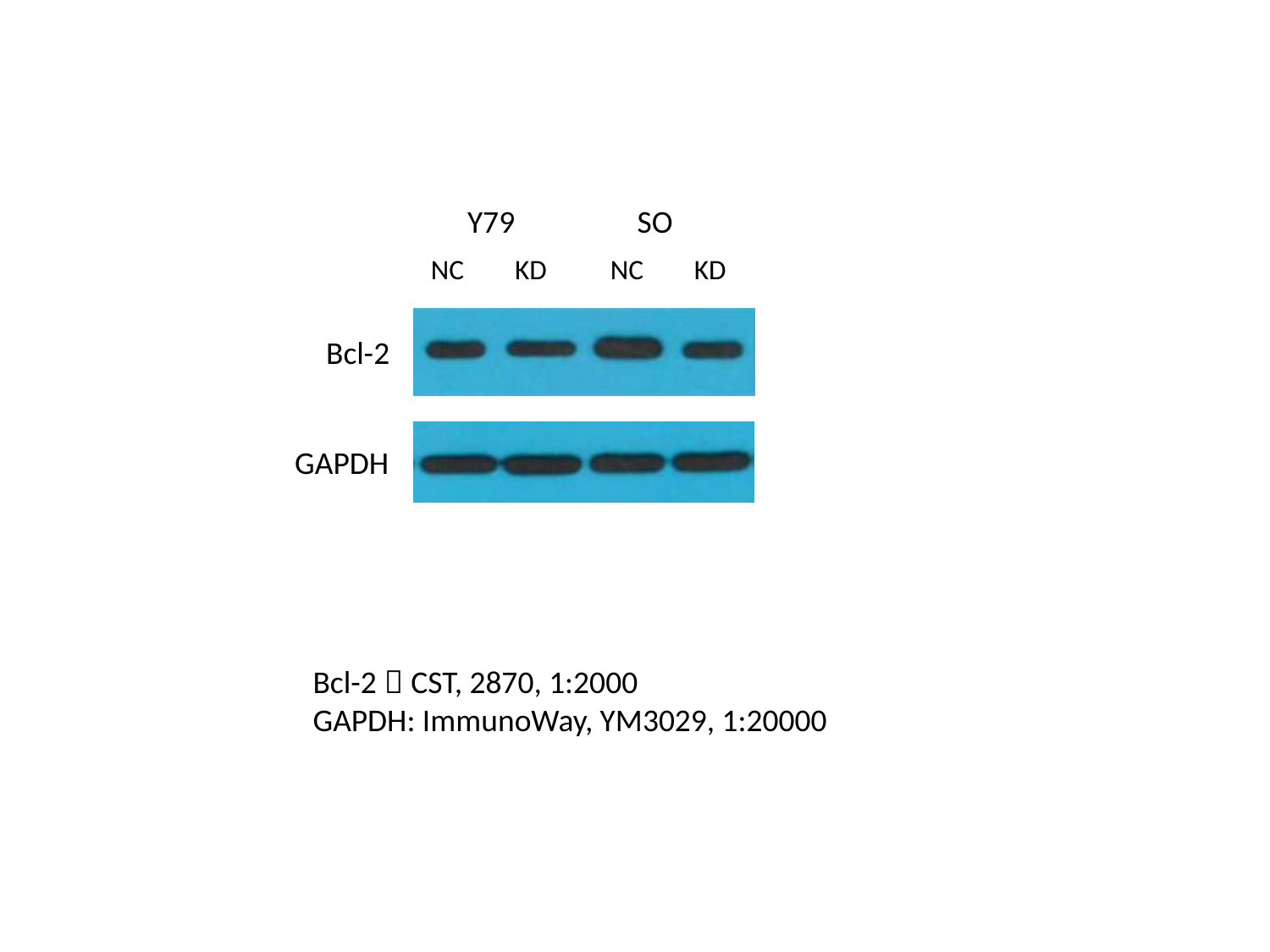

SO
Y79
NC KD NC KD
Bcl-2
GAPDH
Bcl-2：CST, 2870, 1:2000
GAPDH: ImmunoWay, YM3029, 1:20000

## Slide 2
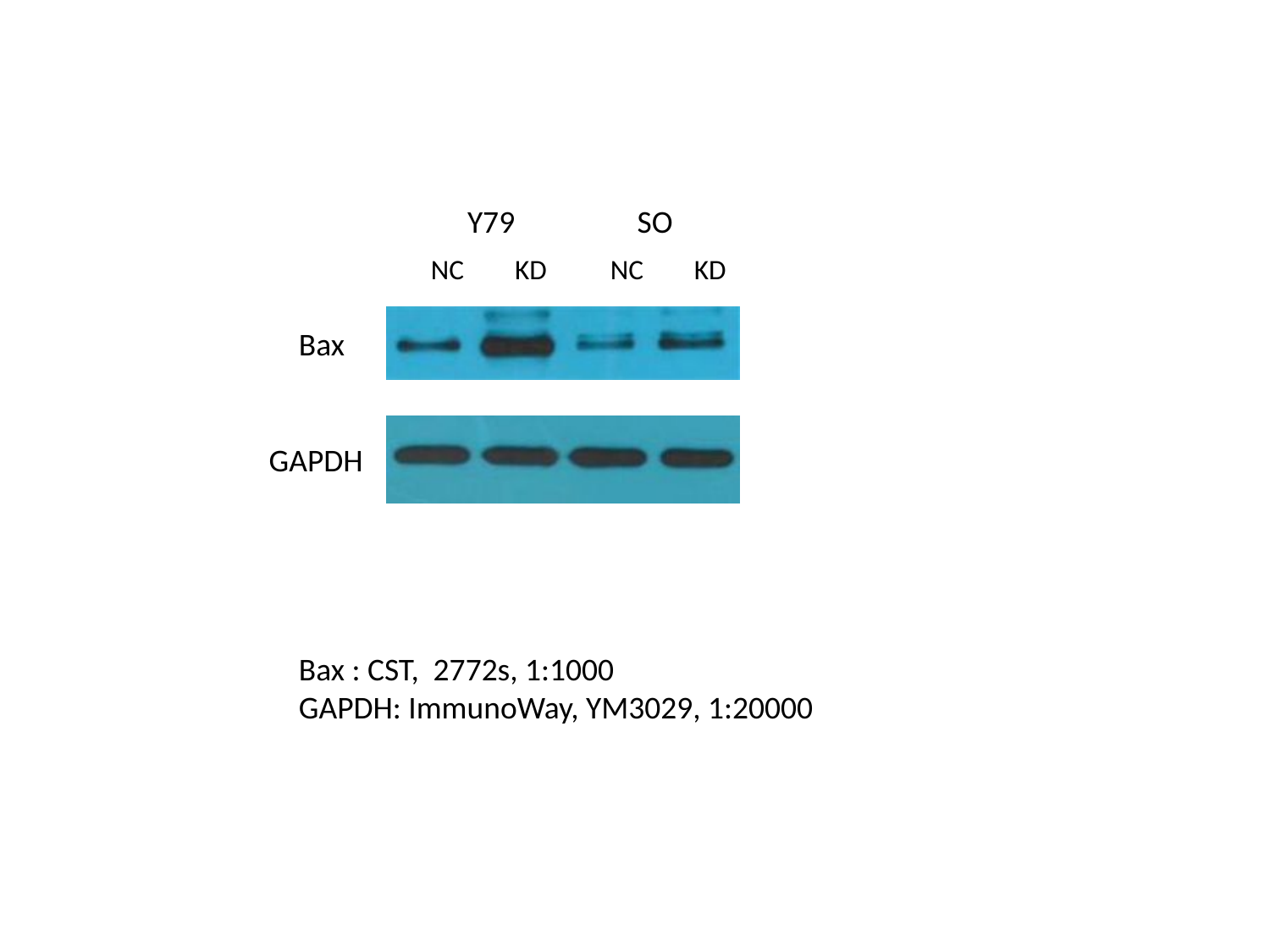

SO
Y79
NC KD NC KD
Bax
GAPDH
Bax : CST, 2772s, 1:1000
GAPDH: ImmunoWay, YM3029, 1:20000

## Slide 3
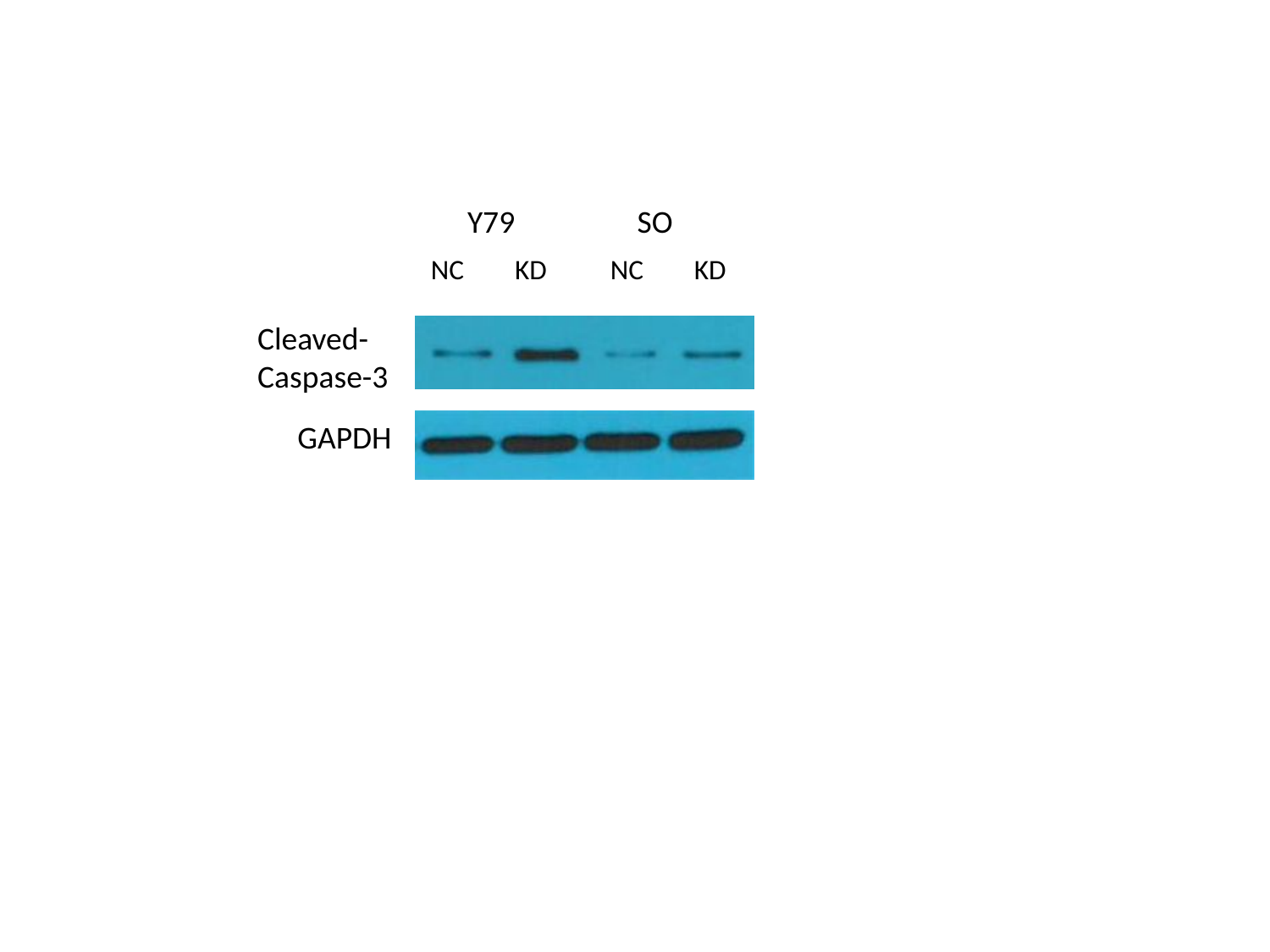

SO
Y79
NC KD NC KD
Cleaved-Caspase-3
GAPDH

## Slide 4
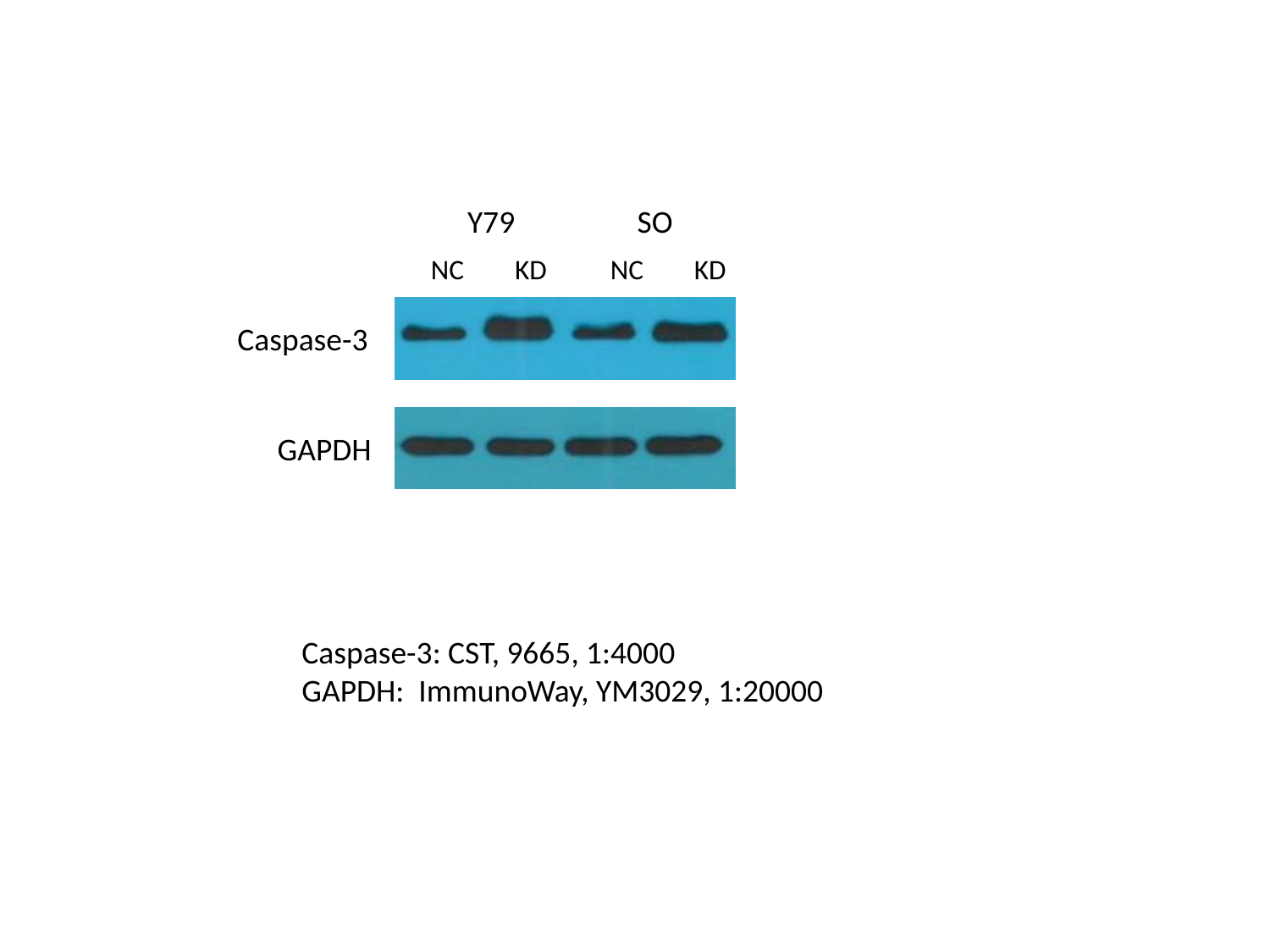

SO
Y79
NC KD NC KD
Caspase-3
GAPDH
Caspase-3: CST, 9665, 1:4000
GAPDH: ImmunoWay, YM3029, 1:20000
