# Supplementary material for: Correction: Lentivirus-Mediated Knockdown of Astrocyte Elevated Gene-1 Inhibits Growth and Induces Apoptosis through MAPK Pathways in Human Retinoblastoma Cells
Source: PLoS One. 2019 Oct 14;14(10):e0223818. doi: 10.1371/journal.pone.0223818 (PMC6791552; doi:10.1371/journal.pone.0223818)

Fig 1: immunohistochemical staining images for 54 patient samples

Please see the attachment for further information

Fig 2: unaltered uncropped Western blot;  RT-PCR individual-level data

Western blot:


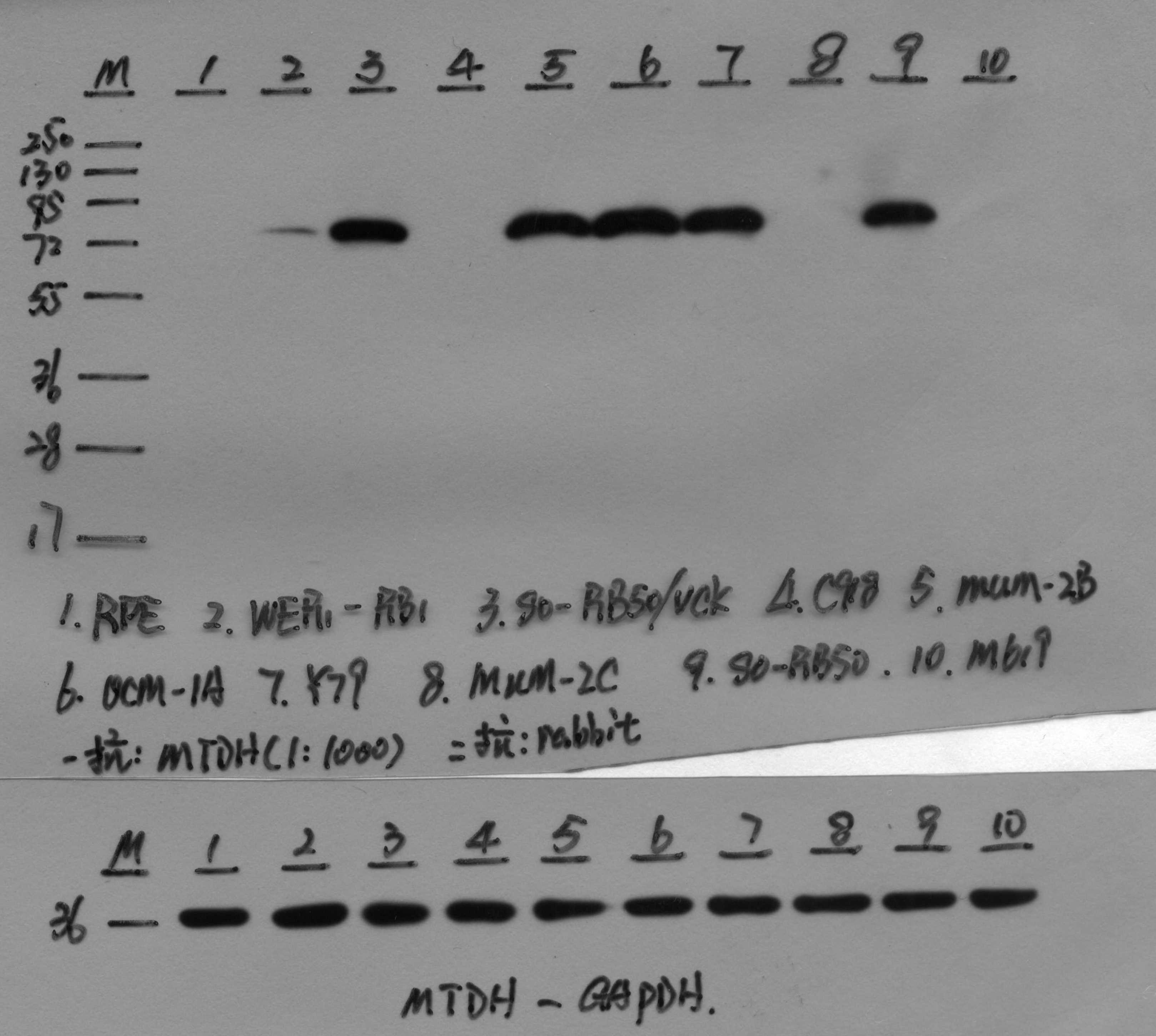

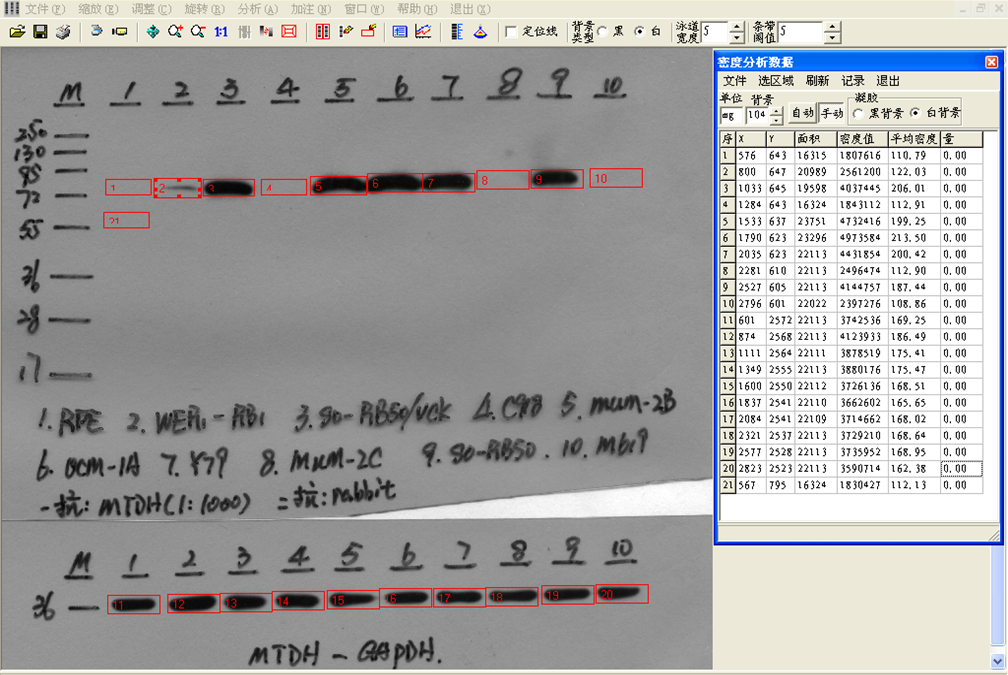


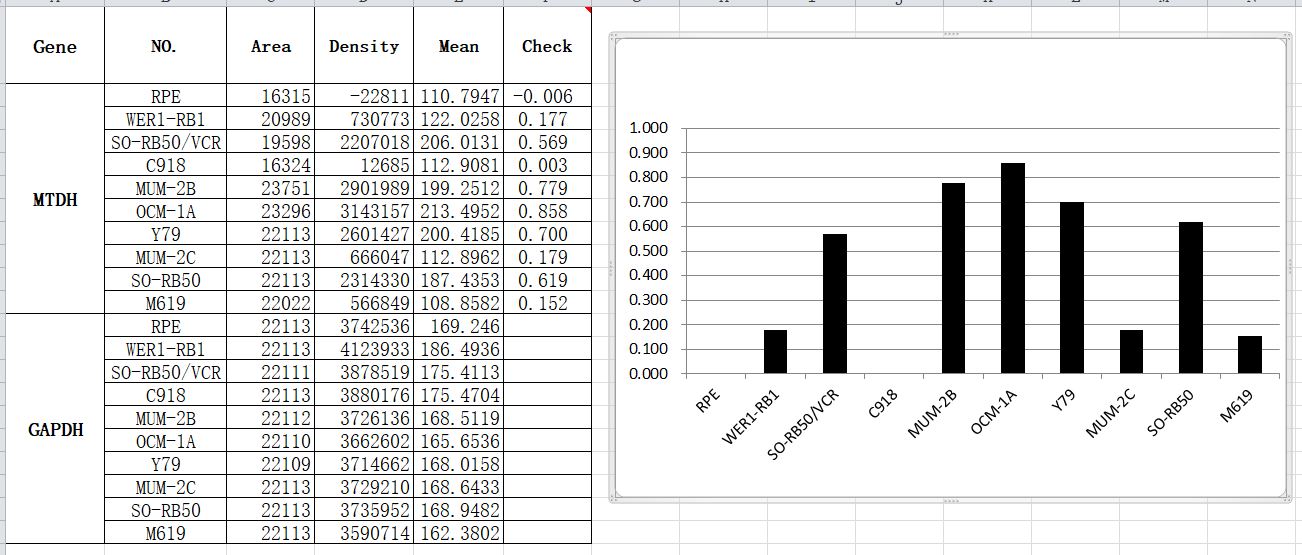


RT-PCR:


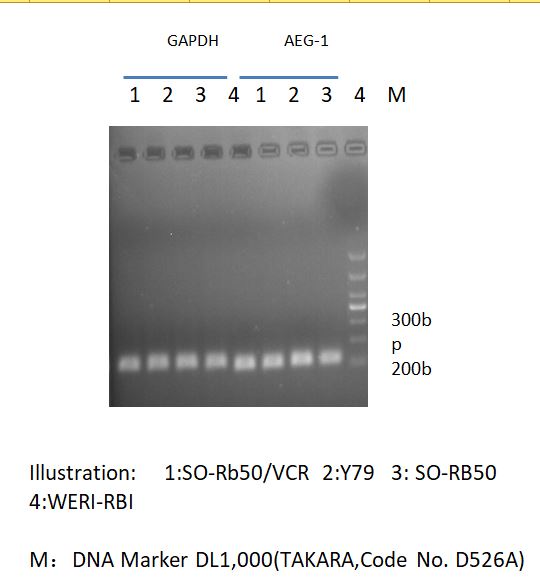


| **Cells** | **Repeat No.** | **Target gene** | **GAPDH Ct** | **MTDH Ct** | **Delta Ct** |
| --- | --- | --- | --- | --- | --- |
| Y79 | 1 | MTDH | 13.06 | 19.11 | 6.05 |
| Y79 | 2 | MTDH | 13.09 | 19.13 | 6.04 |
| Y79 | 3 | MTDH | 13.07 | 19.14 | 6.07 |
| SO-RB50 | 1 | MTDH | 13.15 | 19.23 | 6.08 |
| SO-RB50 | 2 | MTDH | 13.13 | 19.23 | 6.10 |
| SO-RB50 | 3 | MTDH | 13.14 | 19.23 | 6.09 |
| WER1-RB1 | 1 | MTDH | 15.36 | 19.38 | 4.02 |
| WER1-RB1 | 2 | MTDH | 15.28 | 19.30 | 4.02 |
| WER1-RB1 | 3 | MTDH | 15.35 | 19.34 | 3.99 |
| SO-Rb50/VCR | 1 | MTDH | 13.07 | 19.15 | 6.08 |
| SO-Rb50/VCR | 2 | MTDH | 13.05 | 19.18 | 6.13 |
| SO-Rb50/VCR | 3 | MTDH | 13.07 | 19.17 | 6.10 |

Fig 3: microscopy images; RT PCR individual-level data; uncropped, unaltered Western blot file

The microscopy images need check out the attachment for more details

**The date of PCR in knock-down of *AEG-1* in SO-Rb50**

|  | *GAPDH* | *AEG-1* | ∆Ct | -∆∆Ct | 2^-∆∆Ct^ | average | Std. | P | efficiency |
| --- | --- | --- | --- | --- | --- | --- | --- | --- | --- |
| NC | 14.28 | 23.26 | 8.98 | -0.073 | 0.950 | 1.001 | 0.052 |  |  |
|  | 14.4 | 23.23 | 8.83 | 0.077 | 1.055 |  |  |  |  |
|  | 14.4 | 23.31 | 8.91 | -0.003 | 0.998 |  |  |  |  |
| KD | 16.86 | 26.58 | 9.72 | -0.813 | 0.569 | 0.391 | 0.159 | 0.015 | 0.609 |
|  | 16.51 | 26.96 | 10.45 | -1.543 | 0.343 |  |  |  |  |
|  | 16.16 | 27 | 10.84 | -1.933 | 0.262 |  |  |  |  |

**The Western blot in knock-down of *AEG-1* in SO-Rb50**


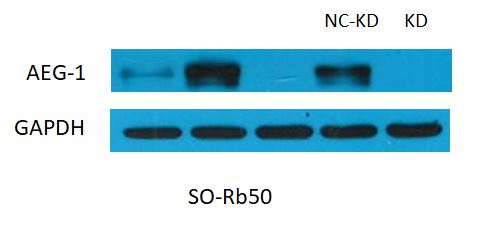


**The date of PCR in knock-down of *AEG-1* in Y79**

|  | *GAPDH* | *AEG-1* | ∆Ct | -∆∆Ct | 2^-∆∆Ct^ | average | Std. | P | efficiency |
| --- | --- | --- | --- | --- | --- | --- | --- | --- | --- |
| NC | 15.74 | 25.21 | 9.47 | -0.053 | 0.964 | 1.001 | 0.059 |  |  |
|  | 15.73 | 25.19 | 9.46 | -0.043 | 0.970 |  |  |  |  |
|  | 15.78 | 25.10 | 9.32 | 0.097 | 1.069 |  |  |  |  |
| KD | 14.66 | 25.20 | 10.54 | -1.123 | 0.459 | 0.459 | 0.006 | 0.004 | 0.541 |
|  | 14.71 | 25.23 | 10.52 | -1.103 | 0.465 |  |  |  |  |
|  | 14.70 | 25.26 | 10.56 | -1.143 | 0.453 |  |  |  |  |

**The Western blot in knock-down of *AEG-1* in Y79**


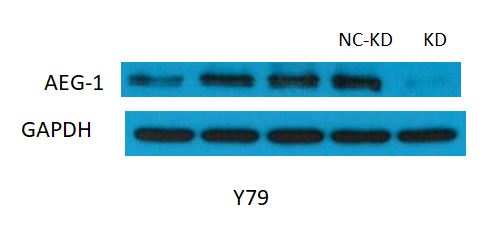


Fig 4: MTT assay individual level chart data from 3 independent experiments; Annexin V flow cytometry raw data (.fcs files) and plotted outputs from 3 independent experimental replicates, and associated individual-level chart data

MTT assay in SO-Rb50：

**The date of OD49 and OD 490 /fold in SORB50**

|  | Time | NC | | | KD | | |
| --- | --- | --- | --- | --- | --- | --- | --- |
| OD 490 | Day 1 | 0.174 | 0.177 | 0.183 | 0.190 | 0.193 | 0.184 |
|  | Day 2 | 0.281 | 0.285 | 0.286 | 0.264 | 0.263 | 0.257 |
|  | Day 3 | 0.618 | 0.673 | 0.660 | 0.423 | 0.479 | 0.475 |
|  | Day 4 | 0.802 | 0.832 | 0.813 | 0.601 | 0.585 | 0.551 |
|  | Day 5 | 0.944 | 0.984 | 0.884 | 0.741 | 0.761 | 0.789 |
| OD 490 /fold | Day 1 | 0.977 | 0.995 | 1.028 | 1.006 | 1.022 | 0.972 |
|  | Day 2 | 1.580 | 1.603 | 1.608 | 1.400 | 1.393 | 1.363 |
|  | Day 3 | 3.469 | 3.781 | 3.707 | 2.237 | 2.538 | 2.513 |
|  | Day 4 | 4.503 | 4.672 | 4.566 | 3.183 | 3.096 | 2.918 |
|  | Day 5 | 5.305 | 5.530 | 4.965 | 3.923 | 4.031 | 4.179 |

**The average and standard deviation of OD49and OD 490 /fold in SORB50**

|  | | OD490 | | OD490/fold | |
| --- | --- | --- | --- | --- | --- |
|  |  | NC | KD | NC | KD |
| Average | Day 1 | 0.178 | 0.189 | 1.000 | 1.000 |
|  | Day 2 | 0.284 | 0.262 | 1.597 | 1.385 |
|  | Day 3 | 0.650 | 0.459 | 3.652 | 2.429 |
|  | Day 4 | 0.815 | 0.579 | 4.580 | 3.066 |
|  | Day 5 | 0.938 | 0.764 | 5.266 | 4.045 |
| STD. | Day 1 | 0.005 | 0.005 | 0.026 | 0.025 |
|  | Day 2 | 0.003 | 0.004 | 0.015 | 0.020 |
|  | Day 3 | 0.029 | 0.032 | 0.163 | 0.167 |
|  | Day 4 | 0.015 | 0.026 | 0.085 | 0.135 |
|  | Day 5 | 0.051 | 0.024 | 0.285 | 0.129 |

Annexin V flow cytometry raw data in SO-Rb50：


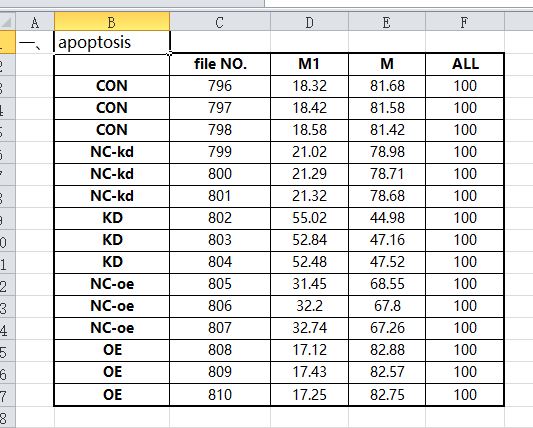


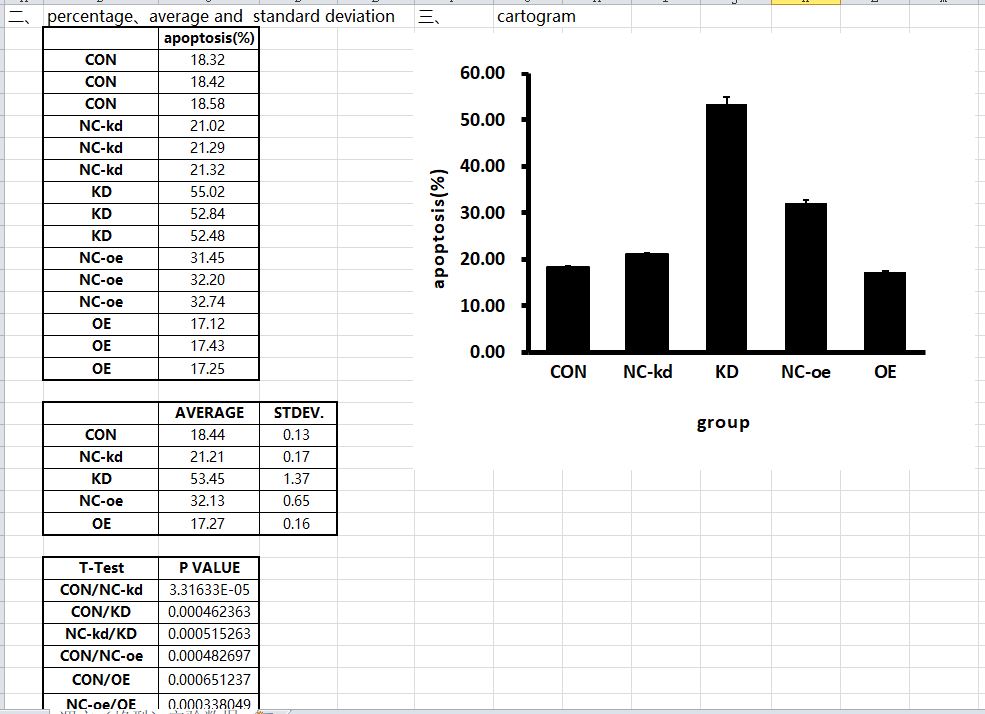


MTT assay inY79：

**The date of OD49and OD 490 /fold in Y79**

|  | Time | NC | | | KD | | |
| --- | --- | --- | --- | --- | --- | --- | --- |
| OD 490 | Day 1 | 0.171 | 0.169 | 0.165 | 0.170 | 0.169 | 0.168 |
|  | Day 2 | 0.235 | 0.238 | 0.239 | 0.227 | 0.229 | 0.228 |
|  | Day 3 | 0.422 | 0.447 | 0.425 | 0.398 | 0.383 | 0.386 |
|  | Day 4 | 0.563 | 0.581 | 0.544 | 0.499 | 0.524 | 0.502 |
|  | Day 5 | 0.749 | 0.771 | 0.732 | 0.643 | 0.661 | 0.659 |
| OD 490 /fold | Day 1 | 1.016 | 1.004 | 0.980 | 1.005 | 1.003 | 0.992 |
|  | Day 2 | 1.396 | 1.410 | 1.419 | 1.343 | 1.354 | 1.350 |
|  | Day 3 | 2.501 | 2.647 | 2.520 | 2.356 | 2.272 | 2.288 |
|  | Day 4 | 3.337 | 3.444 | 3.223 | 2.954 | 3.105 | 2.972 |
|  | Day 5 | 4.440 | 4.571 | 4.341 | 3.808 | 3.918 | 3.906 |

**The average and standard deviation of OD49and OD 490 /fold in Y79**

|  | | OD490 | | OD490/fold | |
| --- | --- | --- | --- | --- | --- |
|  |  | NC | KD | NC | KD |
| Average | Day 1 | 0.169 | 0.169 | 1.000 | 1.000 |
|  | Day 2 | 0.238 | 0.228 | 1.408 | 1.349 |
|  | Day 3 | 0.431 | 0.389 | 2.556 | 2.305 |
|  | Day 4 | 0.562 | 0.508 | 3.335 | 3.010 |
|  | Day 5 | 0.751 | 0.654 | 4.451 | 3.877 |
| STD. | Day 1 | 0.003 | 0.001 | 0.018 | 0.007 |
|  | Day 2 | 0.002 | 0.001 | 0.011 | 0.006 |
|  | Day 3 | 0.013 | 0.008 | 0.080 | 0.045 |
|  | Day 4 | 0.019 | 0.014 | 0.111 | 0.083 |
|  | Day 5 | 0.019 | 0.010 | 0.115 | 0.060 |

Annexin V flow cytometry raw data inY79：


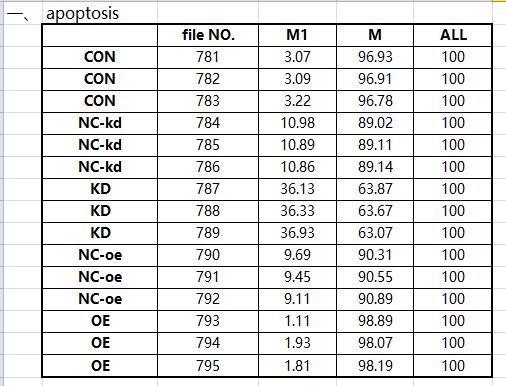


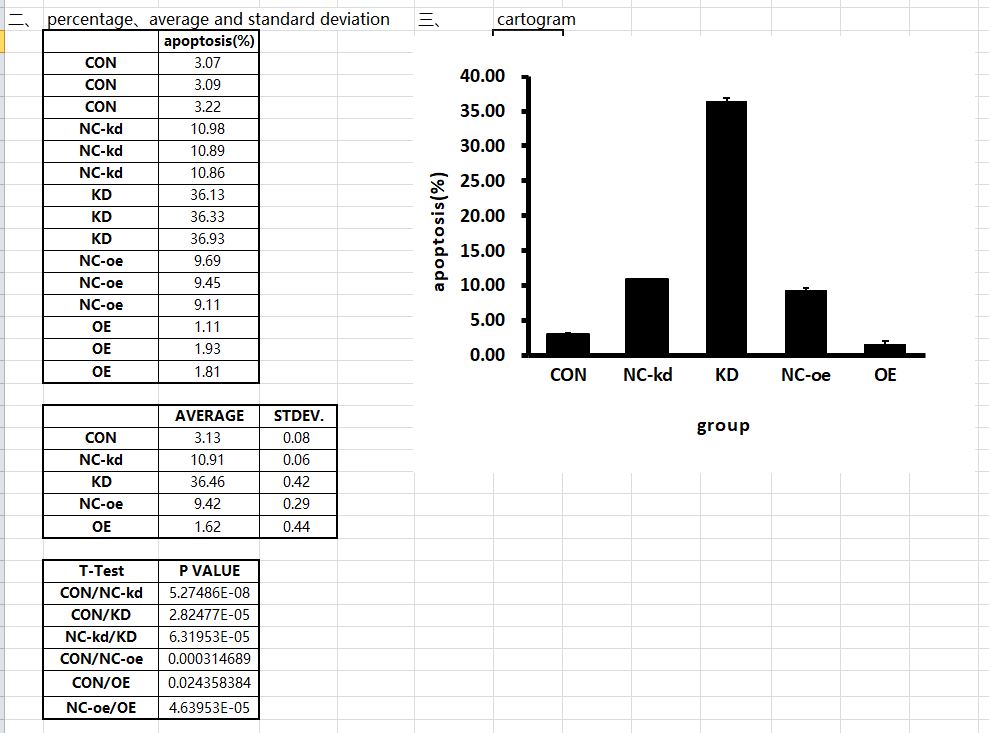


Fig 5: uncropped, unaltered Western blot image files


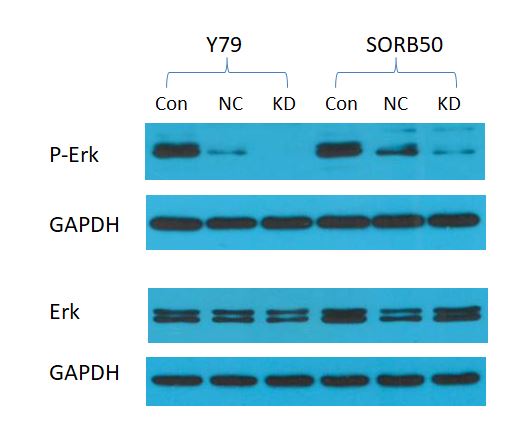


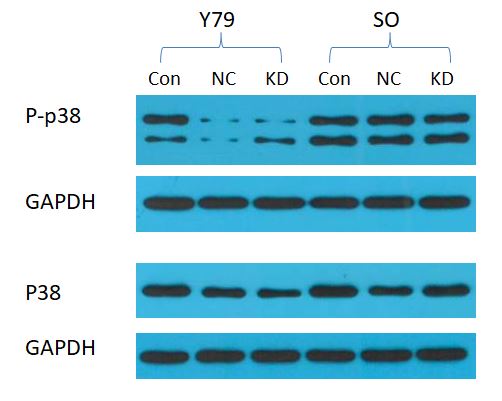


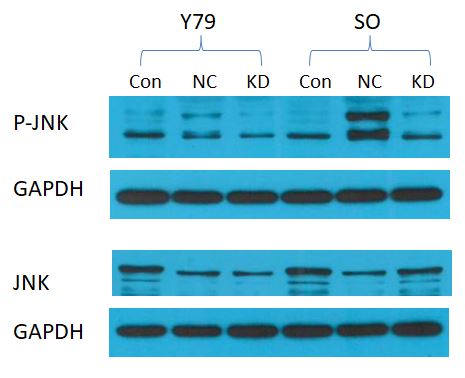


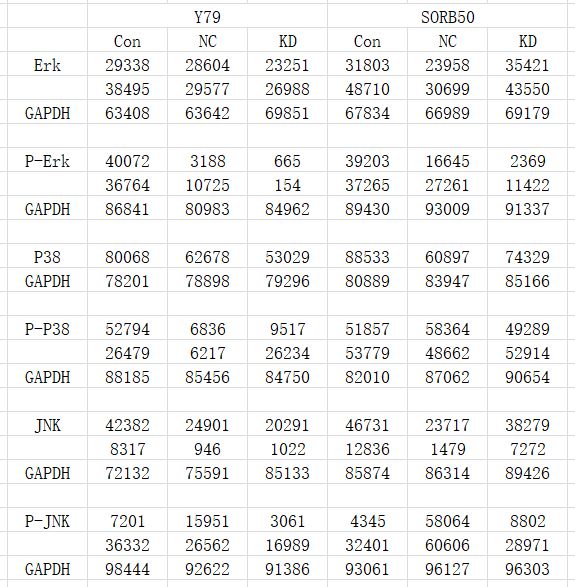

Supplement: S1 File — (ZIP) [file pone.0223818.s001.zip › Original data.docx]
