# Supplementary figures and images for: Correction: Lentivirus-Mediated Knockdown of Astrocyte Elevated Gene-1 Inhibits Growth and Induces Apoptosis through MAPK Pathways in Human Retinoblastoma Cells
Source: PLoS One. 2019 Oct 14;14(10):e0223818. doi: 10.1371/journal.pone.0223818 (PMC6791552; doi:10.1371/journal.pone.0223818)

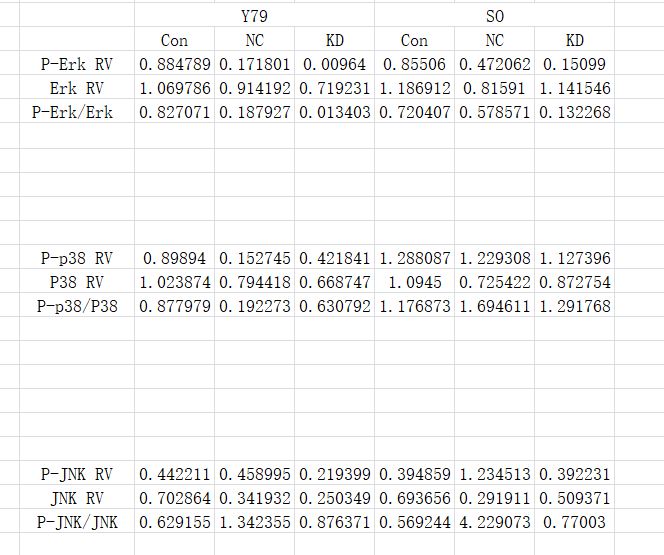

Supplement: S1 File — (ZIP) [file pone.0223818.s001.zip › Fig 5B_data.jpg]

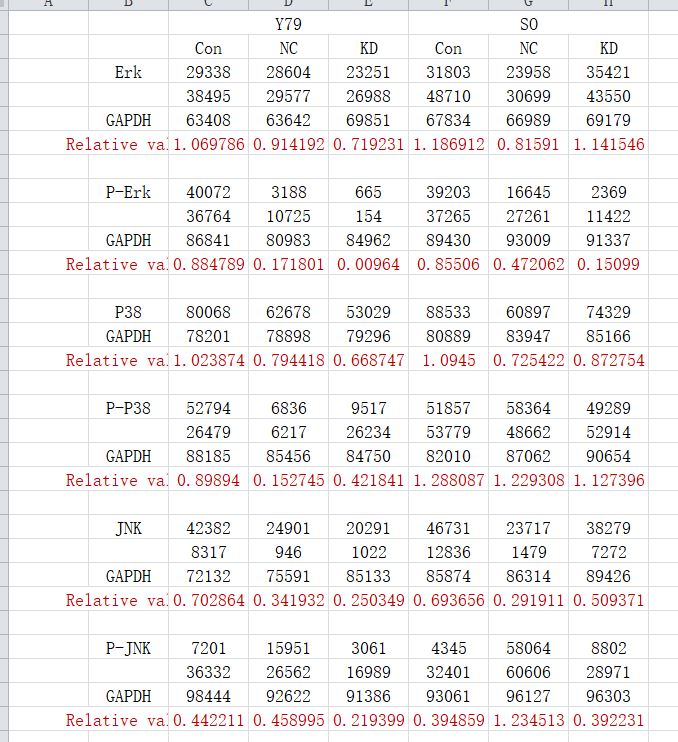

Supplement: S1 File — (ZIP) [file pone.0223818.s001.zip › Fig 5B_readings.jpg]
